# Tracking SARS-CoV-2 Omicron lineages using real-time reverse transcriptase PCR assays and prospective comparison with genome sequencing

**DOI:** 10.1038/s41598-023-44796-y

**Published:** 2023-10-14

**Authors:** Nathan Zelyas, Kanti Pabbaraju, Matthew A. Croxen, Tarah Lynch, Emily McCullough, Stephanie A. Murphy, Sandy Shokoples, Anita Wong, Jamil N. Kanji, Graham Tipples

**Affiliations:** 1 Alberta Precision Laboratories, Public Health Laboratory, Edmonton, AB Canada; 2 Department of Laboratory Medicine and Pathology, University of Alberta, Edmonton, AB Canada; 3 Alberta Precision Laboratories, Public Health Laboratory, Calgary, AB Canada; 4 Department of Pathology and Laboratory Medicine, University of Calgary, Calgary, AB Canada; 5 National Microbiology Laboratory, Public Health Agency of Canada, Edmonton, AB Canada; 6 Division of Infectious Diseases, Department of Medicine, University of Calgary, Calgary, AB Canada; 7 Li Ka Shing Institute of Virology, University of Alberta, Edmonton, AB Canada; 8 Department of Medical Microbiology and Immunology, University of Alberta, Edmonton, AB Canada

**Keywords:** SARS-CoV-2, Clinical microbiology, Infectious-disease diagnostics

## Abstract

Omicron has become the dominant SARS-CoV-2 variant globally since December 2021, with distinct waves being associated with separate Omicron sublineages. Rapid detection of BA.1, BA.2, BA.4, and BA.5 was accomplished in the province of Alberta, Canada, through the design and implementation of real-time reverse transcriptase PCR assays targeting S:N501Y, S:ins214EPE, S:H69/V70, ORF7b:L11F, and M:D3N. Using the combination of results for each of these markers, samples could be designated as belonging to sublineages within BA.1, BA.2, BA.4, or BA.5. The analytical sensitivity of these markers ranged from 132 to 2229 copies/mL and in-laboratory accuracy was 98.9–100%. A 97.3% agreement using 12,592 specimens was demonstrated for the assays compared to genome sequencing. The use of these assays, combined with genome sequencing, facilitated the surveillance of SARS-CoV-2 lineages throughout a BA.5-dominated period.

## Introduction

Since 2021, the appearance of a new SARS-CoV-2 variant of concern (VOC) typically heralds the arrival of a new wave of infections^1–3^. Detection of new VOCs is key in identifying when an increase in infections is expected and whether consideration is warranted for implementing public health measures to curtail the impact of these infections. Lineage surveillance is also needed to determine the continued effectiveness of pharmaceuticals (such as monoclonal antibodies) when specific lineages are known to exhibit resistance and to ensure diagnostic assays are still able to detect the virus^4–6^.

Genome sequencing and real-time reverse transcriptase PCR (rRT-PCR) assays that target single nucleotide polymorphisms (SNPs), deletions, or insertions have been used extensively throughout the COVID-19 pandemic to identify VOCs^7–9^. While genome sequencing allows full genetic characterization of SARS-CoV-2 strains, it requires specialized equipment, technical skills, significant computing power and expertise in analyzing the results. By comparison, rRT-PCR assays provide limited genetic information, but do not have the same technical, monetary, and computing needs as genome sequencing. They can aid in determining variant status and have the advantage of a quicker turn-around-time (a few hours compared to several days or weeks), allowing a more up to date understanding of circulating variants. Many rRT-PCR assays for VOC detection have been described in the literature^10–18^.

While Omicron was declared a VOC by the WHO soon after its detection in multiple countries in late November 2021^19^, there are numerous sublineages of Omicron that have demonstrated enhanced transmissibility and created their own associated waves of infection^1,20,21^. BA.1 caused the initial Omicron wave, followed by BA.2 and then BA.5; currently, areas throughout the world are experiencing waves due to BQ.1.1 or XBB.1.5^1–3^. In the province of Alberta, Canada, rRT-PCR assays have been used since the first VOC, Alpha, was declared^22,23^. To track the major Omicron lineages, new rRT-PCR assays were developed, evaluated, and implemented in Alberta during the summer of 2022 and their performance was compared with genome sequencing.

## Methods

### Population and clinical samples

The province of Alberta has a population of approximately 4.4 million. During July 19 to December 31, 2022 (the period of this study), molecular testing for SARS-CoV-2 was performed for individuals at risk for severe illness who would benefit from early treatment with antivirals, healthcare workers, workers in high-risk settings, patients seen in an emergency department, and inpatients^24^. Upper respiratory tract specimens including nasopharyngeal (NP) and throat swabs were collected and transported in Universal Transport Medium (COPAN Diagnostics) or Viral Transport Medium (Yocon). Other specimen types included NP aspirate and lower respiratory tract samples, such as bronchoalveolar lavage, bronchial wash, and endotracheal tube aspirate. SARS-CoV-2 testing was carried out using several different platforms, depending on the local laboratory (Table S1). All samples found to be SARS-CoV-2 positive were sent to the provincial public health laboratories for testing with Omicron rRT-PCR assays and a subset were also tested by genome sequencing. This study was carried out in accordance with local guidelines and regulations and approved by the University of Alberta Human Research Ethics Board (reference number Por00108722). The Research Ethics Board determined that participant informed consent was not required for this study given that the testing was done as part of routine SARS-CoV-2 public health surveillance.

### Omicron rRT-PCR assays

Nucleic acid extraction from all clinical specimens was performed either on the easyMAG platform (bioMérieux, Marcy-l’Étoile, France) with associated reagents or the MagMAX Express 96 or KingFisher Flex automated extraction and purification systems (Thermo Fisher Scientific, Waltham, USA) with the MagMAX-96 Viral RNA isolation kit (Thermo Fisher Scientific) using input and output volumes of 200 μL and 110 μL, respectively. Primers and probes were designed to target the following mutations: S:ins214EPE, orf7b:L11F, and M:D3N (Table S2). Oligonucleotide sequences that have been described in previous studies were used to target a generic E gene marker, S:N501Y, S:H69/V70, and MS2 bacteriophage as an internal control (Table S2)^22,23,25,26^. Two multiplex assays (hereafter referred to as the Omicron assays) were designed: one targeting an E gene region, S:N501Y, S:ins214EPE, and MS2; and the other targeting S:H69/V70, orf7b:L11F, and M:D3N. The primer and probe sequences with final concentrations used are indicated in Table S2. Primers were obtained from LGC Biosearch Technologies (Petaluma, CA, USA) and probes from Applied Biosystems (ABI; Foster City, CA, USA). The assays were each run using TaqMan Fast Virus One-Step RT-PCR Master Mix (ABI) and 5 μL of extracted template. Reverse transcription was carried out at 50 °C for 5 min followed by incubation at 95°C for 20 s; real-time PCR was then carried out with 45 cycles of 95 °C for 3 s followed by 60 °C for 30 s using the 7500 Fast real-time PCR system (ABI). During November 23-December 31, 2022, specimens were run using the above assays. Prior those dates, ORF7b:L11F was run using a different fluorophore and M:D3N had minor differences in oligonucleotide concentrations; these differences are noted in Table S2.

### Omicron assay evaluation

**Figure 1:**
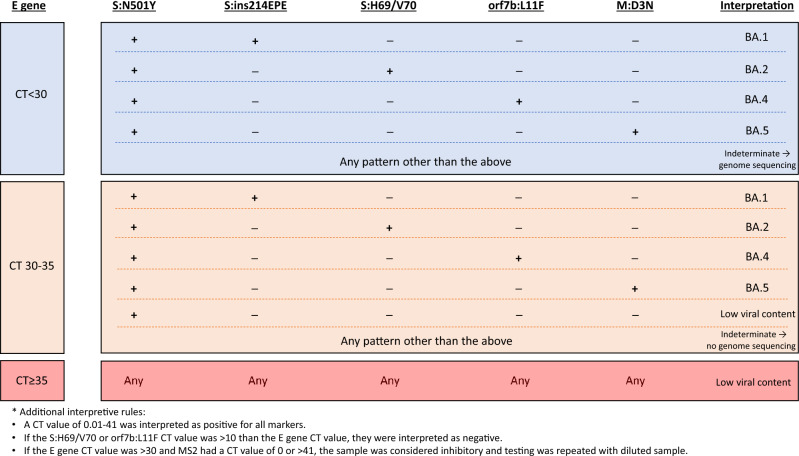
Interpretation of the Omicron assays.

Both Omicron assays were evaluated for each target’s analytical sensitivity, analytical specificity, inter- and intra-assay reproducibility, and accuracy. Analytical sensitivity was determined using in vitro RNA derived from wild-type virus (E gene and S:H69/V70 markers), the Alpha variant (S:N501Y marker), or synthetic oligonucleotides (S:ins214EPE, ORF7b:L11F, and M:D3N markers). The areas spanned by the assays were amplified by PCR and cloned using the TOPO TA Cloning Dual Promoter kit (Life Technologies, CA, USA). Transcription to produce in vitro RNA was performed using the T7 RiboMAX Express (Promega, Madison, WI, USA) or RiboMAX SP6 RNA Production System (Promega). RNA was quantified using a spectrophotometer. Analytical sensitivity was determined by testing tenfold serial dilutions of the quantified RNA in nine replicates followed by probit analysis to calculate the 95% limit of detection. Analytical specificity was determined by testing a panel of common and/or related respiratory pathogens (influenza A and B, respiratory syncytial virus, parainfluenza 3, coxsackievirus B6, human rhinovirus 1b, human adenovirus type 10, human metapneumovirus-2, bocavirus, endemic coronaviruses [229E, NL63, OC43, HKU1], MERS-CoV, SARS-CoV-1, *Mycoplasma pneumoniae*, *Chlamydia pneumoniae*, *Legionella pneumophila*, *Bordetella pertussis*, *Hemophilus influenzae*, *Streptococcus pneumoniae*, and *Neisseria meningitidis*). Inter- and intra-assay reproducibility was assessed by running clinical samples positive for BA.1, BA.2, BA.4, or BA.5 with high (cycle threshold [CT] values of ~ 20) and low (CT values of ~ 30) viral RNA concentrations. The percent coefficient of variation, based on CT values, was calculated for each marker. Accuracy was determined by running a panel of 280 clinical samples with known lineages based on genome sequencing on both Omicron assays. The accuracy panel consisted of 9 samples containing BA.1 sublineages, 57 containing BA.2 and its sublineages, 75 containing BA.4 and its sublineages, 121 containing BA.5 and its sublineages, and 18 containing non-Omicron lineages (Alpha3, Beta3, Gamma2, Delta4, A.23.11, B.1.1.3181, B.1.1.5191, B.1.361, and B.1.4272). The detection of S:N501Y plus one other Omicron lineage-specific marker was interpreted as either BA.1 (S:ins214EPE), BA.2 (S:H69/V70), BA.4 (ORF7b:L11F), or BA.5 (M:D3N). The CT value for the E gene was used to assess the viral RNA content. At lower RNA levels, the Omicron assay markers were less reliable and such samples were left as uninterpretable. Bacteriophage MS2 was spiked into all primary specimens as an extraction and inhibition control. Due to observed cross-reactivity of the S:H69/V70 and ORF7b:L11F markers, a rule was implemented that these probes would be considered negative if the CT values of the markers were > 10 above the CT value of the E gene marker. The interpretation algorithm is demonstrated in Fig. 1.

### Genome sequencing

Full genome sequencing was performed on a subset of SARS-CoV-2 positive specimens. Specimens with an E gene CT value over 30 were deemed too low in viral content to sequence and were usually excluded. The full genome was amplified as 1.2 kb amplicons using the Freed primer scheme^27^. Library preparation and sequencing was carried out using either Oxford Nanopore Technologies (ONT) (Oxford, UK) or Illumina (San Diego, USA) methodologies. The ARTIC LoCost protocol^28^ and Ligation sequencing kit (SQK-LSK109) were used to prepare Nanopore libraries, which had 15–20 ng loaded onto the FLO-MIN 106D flow cells (ONT) for sequencing. Sequence data using the ONT protocol was compiled with the artic 1.1.3 pipeline^29^. Illumina libraries were prepared with the DNA Prep Kit and sequenced on a MiSeq using the 300 cycle MiSeq Reagent Kit V2 Micro or on a MiniSeq using the MiniSeq Mid Output 300 cycle kit (Illumina). Sequence data using the Illumina protocol was processed with the OICR fork^30^ of the ncov2019-illumina-nf pipeline^31^ and with an updated version using freebayes as the variant caller^32^. Sequence quality was assessed using ncov-qc^33^. Lineages were assigned using pangolin^34^ and mutations detected using nextclade^35^.

### Statistical analysis

Analytical sensitivity was determined by calculating the 95% limit of detection for each marker via probit analysis. Inter- and intra-assay reproducibility were calculated as %CVs from CT values of high and low RNA content samples run in triplicate on three independent runs. Accuracy was calculated as the proportion of Omicron assays’ results agreeing with genome sequencing up to the parent lineage (for example, if a sample was BA.1.1 positive by genome sequencing and using the Omicron assays led to an interpretation of BA.1, this was considered agreement). Seven-day rolling averages for the Omicron assays’ results were determined for each day. For samples that underwent both Omicron assay testing and genome sequencing, positive percent agreement (true positives/[true positives + false negatives] × 100%), negative percent agreement (true negatives/[true negatives + false positives] × 100%), positive predictive value (true positives/[true positives + false positives] × 100%), and negative predictive value (true negatives/[true negatives + false negatives] × 100%) were determined using genome sequencing as the reference method. As was done for the accuracy calculation, agreement was considered to have been achieved when the lineage determined by genome sequencing correctly fell under one of the parent lineages of BA.1, BA.2, BA.4, and BA.5. Statistical analysis was assisted by MedCalc^36^.

## Results

### Omicron assays’ performance

The summary of the performance of the Omicron assays’ markers is shown in Table 1. Each marker within the assays showed a 95% limit of detection under 2500 copies/mL and no unexpected cross-reactivity was detected. The %CV of the markers had a range of 0.22–1.08% for inter-assay reproducibility and 0.01–1.28% for intra-assay reproducibility, which were deemed acceptable based on a threshold of 15%^37^. Accuracy ranged from 98.9 to 100% depending on the marker tested.

**Table 1:** Laboratory evaluation of the Omicron screening assays.

	E gene	S:N501Y	S:ins214EPE	S:H69/V70	ORF7b:L11F	M:D3N
Analytical sensitivity (copies/mL)	633	461	132	211	557	2,229
Analytical specificity (%)	100^1^	100	100	100	100	100
Inter-assay reproducibility (% CV)^2^	0.30–0.48	0.24–0.84	0.22–0.51	0.77–1.08	0.77–1.08	0.45–0.61
Intra-assay reproducibility (% CV)^2^	0.05–0.46	0.06–1.28	0.01–0.72	0.26–0.65	0.14–0.87	0.11–0.80
Accuracy (%)	100	100	99.6	100	98.9	99.6

^1^The generic E gene marker did amplify with SARS-CoV-1 but this is a known and expected reaction^25^.

^2^The coefficient of variation percentage in CT values was used as a measure of reproducibility.

### Prospective comparison of the Omicron assays to genome sequencing

In total, 12,592 SARS-CoV-2 positive specimens were subjected to both genome sequencing and the Omicron assays during the study period with most specimens being positive for BA.5 and its sublineages (Table 2). The Omicron assays showed high levels of agreement and predictive values for all lineages, though the positive percent agreement was lowest for BA.2 and its sublineages (88.1%) and the negative predictive value was lowest for BA.5 and its sublineages (87.4%) (Table 3). The overall agreement between the Omicron assays and genome sequencing was 97.6% (95% CI 97.3–97.8%).

**Table 2:** Comparison of genome sequencing and the Omicron assay results.

	Genome sequencing result					
Omicron screening assay result	BA.1 and sublineages	BA.2 and sublineages	BA.4 and sublineages	BA.5 and sublineages	BA.2 and BA.5 recombinants	Total
BA.1	16	0	0	0	0	16
BA.2	0	458	0	0	15	473
BA.4	0	0	728	0	0	728
BA.5	0	2	0	11,084	0	11,086
Indeterminate^1^	0	55	58	170	4	287
Low viral content	0	1	0	1	0	2
Total	16	516	786	11,255	19	12,592

^1^Indeterminate indicates that the Omicron assays did not provide an interpretable result consistent with a circulating Omicron lineage.

**Table 3:** Performance characteristics of the Omicron assays compared to genome sequencing^1^.

	BA.1 (%)	BA.2 (%)	BA.4 (%)	BA.5 (%)
Positive percent agreement	100 (79.4–100)	88.1 (85.0–90.7)	92.6 (90.6–94.4)	98.3 (98.1–98.5)
Negative percent agreement	100 (99.97–100)	99.9 (99.8–99.9)	100 (99.97–100)	99.9 (99.5–99.98)
Positive predictive value	100	96.8 (94.8–98.1)	100	99.98 (99.9–100)
Negative predictive value	100	99.5 (99.4–99.6)	99.5 (99.4–99.6)	87.4 (85.8–88.9)

^1^95% confidence intervals are displayed in parentheses.

There were 287 specimens that yielded indeterminate results on the Omicron assays where the mutation profile was not consistent with BA.1, BA.2, BA.4 or BA.5. The majority of these (n = 173) had a profile where S:N501Y was detected, but no other lineage-specific marker was detected. This occurred for BA.2 strains with ΔH69/V70, BA.4 strains with deletions in the 27,786 to 27,800 nucleotide positions encompassing ORF7b:L11F probe-binding regions, reactions with atypical ORF7b:L11F curves when the HEX fluorophore was used (this was corrected by using VIC in the later iteration of the assay), and reactions where M:D3N signal did not reach threshold fluorescence (also corrected by increasing primer and probe concentrations in the later version of the assay). Some BA.2 positive samples were negative for S:N501Y (n = 31), the majority of which were attributable to mutations in the region targeted by the 3’ end of the forward primer (G23012A/A23013G, corresponding to S:E484K), which was observed for lineage CM.2 strains. False-positive signal was observed for S:ins214EPE (n = 31; 0.2% of all samples) or ORF7b:L11F (n = 25; 0.2% of all samples) in some samples due to higher levels of background fluorescence of the S:ins214EPE probe or normalized algorithm plots showing ORF7b:L11F signal in the absence of fluorescence in the raw data plot. Fourteen samples were positive for both S:H69/V70 and M:D3N, four of which were found to be XBD, a recombinant of BA.5 and BA.2.75 sublineages^38^. Two BA.5 specimens (both BA.5.2.1) were ORF7b:L11F positive on the Omicron assays, and this was confirmed by sequencing.

645 specimens were successfully tested using the Omicron assays but were unsuccessful when genome sequencing was attempted. For those specimens, the median CT value of the E gene marker was 28.09. This compares to a median CT value of 20.93 for specimens that were successfully sequenced.

### Omicron lineage detection in Alberta

**Figure 2:**
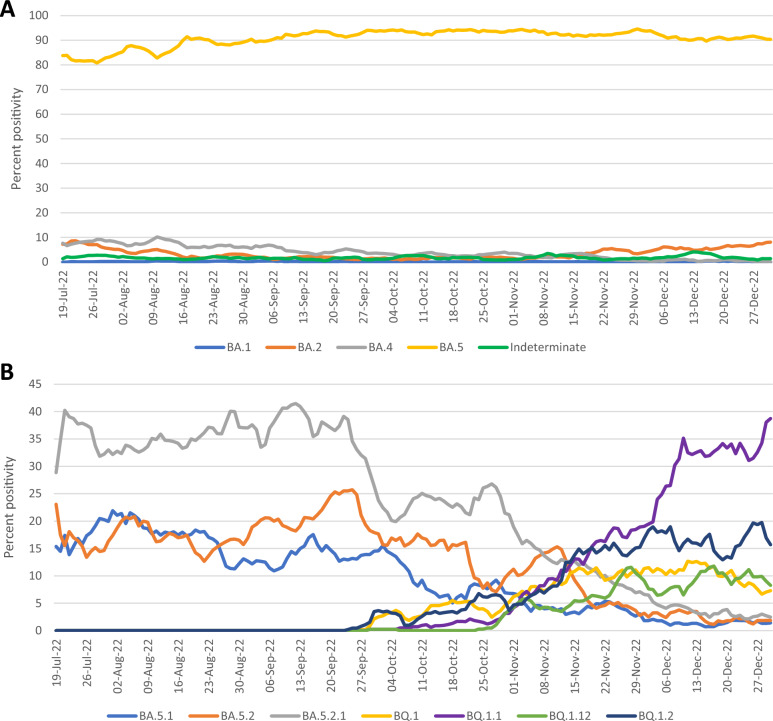
Positivity rates of Omicron lineages. A, Positivity rates of BA.1, BA.2, BA.4, and BA.5 lineages and indeterminate results (those that do not fit the defined BA.1, BA.2, BA.4, or BA.5 profiles) determined by the Omicron assays are shown as rolling 7-day averages. Specimens with uninterpretable results due to low viral load were excluded. B, Positivity rates of the most frequently detected sublineages within BA.5 are shown as rolling 7-day averages. Only BA.5 sublineages were included.

During the study period, 32,429 SARS-CoV-2 positive specimens underwent testing with the Omicron assays. Overall, there were 37 (0.1%) BA.1-positive specimens, 831 (2.6%) BA.2-positive specimens, 1,029 (3.2%) BA.4-positive specimens, and 22,784 (70.3%) BA.5-positive specimens. The Omicron assays did not generate results consistent with circulating Omicron lineages for 443 (1.4%) specimens and 7,305 (22.5%) specimens did not have a high enough viral load to generate an interpretable result. Over time, among the samples with enough viral RNA to interpret the Omicron assay results, the proportion of BA.5-positive samples remained high at 80–95%, while the remaining lineages remained at relatively low levels (Fig. 2A). When examining the sublineages within BA.5 determined by genome sequencing, BA.5.1, BA.5.2, and BA.5.2.1 dominated early during the study period, but were overtaken by BQ.1 and its related lineages starting in mid-November 2022, with BQ.1.1 becoming the most frequently detected lineage by the end of December (Fig. 2B).

## Discussion

The utility of PCR assays targeting specific mutations to identify SARS-CoV-2 variants of concern has been demonstrated by many groups^10–18^. The relatively low complexity in setup and analysis, high throughput, and quick turn-around-time of these types of assays compared to genome sequencing has allowed near real-time tracking of variants for laboratories without sequencing capabilities. The Omicron assays that we implemented locally demonstrated high sensitivity and specificity during the in-laboratory evaluation while also showing a high level of concordance with genome sequencing, indicating that such assays continue to be useful to monitor emerging variants as SARS-CoV-2 evolves.

However, it is important to note that even with the use of these assays, an undercurrent of BA.5 sublineages were competing for dominance beneath the surface of what was detectable using the Omicron assays. The Omicron assays did not provide the resolution necessary to observe this apparent struggle between BA.5 sublineages; it was observable only by using genome sequencing. While it certainly is possible that assays could have been designed and implemented that could detect the major BA.5 sublineages that emerged (BA.5.1, BA.5.2, BA.5.2.1, and BQ.1 and its sublineages), continually deploying new PCR assays whenever a new lineage begins to show an increased growth rate represents a major challenge in such SARS-CoV-2 surveillance programs. These assays require extensive work in their design, evaluation, and implementation; interpretation of the mutation patterns, especially with multiple lineages, emerging recombinants, and overlap in SNPs, can be challenging. Predicting which lineages warrant monitoring in a timely fashion is not always possible. We did not have local capability for the synthesis of oligonucleotide probes, requiring long-distance orders to be completed that took multiple weeks to reach our facility. Even after the reagents are available, there is no guarantee that the assays will function as planned which may cause the need for re-design and re-ordering of oligonucleotides, incurring additional weeks of delay before validation and implementation are possible. Since these assays target very specific regions, there is limited flexibility for probe design if the SNP lies in a region of low complexity or secondary structure.

When considering these challenges and the findings of this study, it appears that SARS-CoV-2 surveillance benefits from a strategy that combines mutation-specific PCR assays and genome sequencing. The Omicron assays demonstrated a higher sensitivity than genome sequencing, based on the number of specimens that were not successfully sequenced but still returned an interpretable result with the Omicron assays. Genome sequencing allows the identification of emerging sublineages which would otherwise go undetected by the Omicron assays and helps to determine which mutations may be worth incorporating into future PCR assays based on local epidemiology. A recent study examining the accuracy of SARS-CoV-2 variant testing across European laboratories found that performance was best in laboratories performing both rRT- PCR assays and sequencing compared to using just one of these methodologies^39^. This is consistent with our findings.

Some groups have used S gene Sanger sequencing as a method to determine the SARS-CoV-2 lineage^40–43^. While this may represent a reasonable intermediate approach when compared to SNP rRT-PCR assays and genome sequencing, this method faces many of the same challenges as genome sequencing: it requires specialized equipment not always available in clinical laboratories, it is laborious and requires sequence analysis, the turnaround time may be prolonged, and it may be more expensive to carry out than rRT-PCR.

Most studies in the published literature describing rRT-PCR assays that identify Omicron lineages are only capable of differentiating the original Omicron lineage (B.1.1.529, equivalent to BA.1) from other VOCs or BA.1 from BA.2^15,44–49^. Few studies have been published which describe rRT-PCR assays that detect and distinguish between BA.1, BA.2, BA.4, and BA.5. Jessen et al.^50^ developed two assays targeting S:ΔH69/V70 and S:L452R to detect these lineages and found that they had near perfect concordance with genome sequencing for 811 clinical specimens tested by both methods. However, these assays were unable to distinguish BA.4 and BA.5^50^. Another assay that distinguishes the four main Omicron lineages targeting S:S371F/S373P/S375F, S:ΔG142/V143/Y144, S:ins214EPE, S:ΔH69/V70 and N:ΔE31/R32/S33, has been described and run as a pentaplex; evaluation of the assay was limited and performed using positive genomic RNA or gBlock material in duplicate for each lineage to confirm inclusivity and exclusivity for each marker^51^.

Our study had several strengths. Perhaps the greatest strength is the large number of specimens that were subjected to both the Omicron assays and genome sequencing, allowing a very robust comparison between these methodologies. The inclusion of a generic E gene marker helped to act as a control to determine whether the viral RNA content was sufficient to interpret the assays accurately. An internal control to monitor for extraction and inhibition was incorporated into one of the assays, adding a further layer of quality control. Multiple mutation markers needed to be positive for each lineage (at least S:N501Y and one other lineage-specific mutation), ensuring that spurious lineage designations were not generated based on just one marker producing signal by itself. The lack of published rRT-PCR assays capable of distinguishing BA.1, BA.2, BA.4, and BA.5 that have undergone extensive evaluation increases the significance of this work.

There were also several limitations in this study. Genome sequencing was not carried out for all samples subjected to the Omicron assays, so both methods could not be compared for all tested samples. There were few specimens positive for BA.1 and BA.2/BA.5 recombinant lineages, necessitating some caution in the interpretation of the results. The positive percent agreements for BA.2 and BA.4 were lower than for the other lineages (88.1% and 92.6%, respectively) due to a high proportion of samples having indeterminate interpretations of the Omicron assay markers. This was predominantly driven by mutations in the genomes of these isolates, interfering with the assays’ detection of markers. However, these indeterminate results were not outright failures of the assays but rather flagged these samples for genome sequencing to derive the correct lineage. If BA.2 or BA.4 were to surpass BA.5 to become dominant lineages, the Omicron assays may require revision to enhance their sensitivity for these markers.

An important issue to address is that the Omicron assays as designed did not detect the rise of different BA.5 sublineages and only showed a sustained plateau of BA.5 being the predominant lineage in the region. This could be alleviated relatively simply by designing new assays that target different genetic polymorphisms observed in BA.5.2.1 and BQ.1.1, for example, though the continual redesign and implementation of new assays is challenging, as already described. Such rRT-PCR assays will always have limited resolution, but they remain useful in that their interpretation will reflect the local epidemiology. For instance, as XBB.1.5 rises and becomes predominant, this will be detectable by a rise in positivity rate for the BA.2-specific marker (S:H69/V70), and will allow tracking of this recombinant lineage–but it will be important to adjust the interpretation of this maker based on the understanding of local genomic surveillance so that its detection is not mistaken for the presence of the BA.2 parent lineage. Similarly, the recent emergence of BA.2.86 would not be directly detected by the Omicron assays as built based on sequence analysis showing the presence of S:N501Y but the lack of the other markers incorporated into the assays^52^. However, a BA.2.86 positive specimen would be interpreted as indeterminate by the assays and route to genome sequencing if the viral load was sufficient.

The combined use of rRT-PCR assays targeting specific key mutations and genome sequencing continues to be a useful approach for the molecular surveillance of SARS-CoV-2. Future work should focus on easing the challenges inherent in creating and operationalizing new mutation-specific rRT-PCR assays so that they can be implemented quicker as lineages quickly rise and fall. This could be accomplished in a variety of ways, such as implementing local or in-house primer and probe manufacturing capabilities, establishing global networks for the sharing of reagents, and strengthening partnerships with existing commercial manufacturers. As well, further studies are warranted to enable genome sequencing to be faster, cheaper, and more technically accessible–such efforts could alleviate any need for variant rRT-PCR assays in the future.

### Supplementary Information

Supplementary Information.

Supplementary Table S3.

## Data Availability

The genome sequencing data generated and analyzed during this study are available in the GISAID repository at https://gisaid.org/ and accession numbers for sequences successfully uploaded to GISAID are indicated in Table S3 (some genome sequences were not accepted by GISAID due to the presence of frameshifts or not submitted if coverage was less than 90%—this sequence data can be obtained from the corresponding author on reasonable request). Raw data generated by the Omicron assays are not publicly available but can be obtained from the corresponding author on reasonable request.
