# Supplementary material for: Tracking SARS-CoV-2 Omicron lineages using real-time reverse transcriptase PCR assays and prospective comparison with genome sequencing
Source: Sci Rep. 2023 Oct 14;13:17478. doi: 10.1038/s41598-023-44796-y (PMC10576821; doi:10.1038/s41598-023-44796-y)
Supplement: Supplementary file 1 — Supplementary Information. [file 41598_2023_44796_MOESM1_ESM.docx]

**Supplementary Material**

**Table S1. COVID-19 nucleic acid tests used in Alberta during July 19-December 31, 2022.**

| **Assay** | **Target(s)** | **Manufacturer or reference** |
| --- | --- | --- |
| cobas SARS-CoV-2 | orf1ab and E gene | Roche |
| Allplex 2019-nCoV assay | N, E, and RdRP genes | Seegene |
| Simplexa COVID-19 Direct | orf1ab and S gene | DiaSorin |
| Xpert Xpress SARS-CoV-2 | N2 region and E gene | Cepheid |
| Xpert Xpress SARS-CoV-2/Flu/RSV | N2 region and E gene | Cepheid |
| Xpert Xpress SARS-CoV-2/Flu/RSV *plus* | N2 region, E and RdRP genes | Cepheid |
| Aptima SARS-CoV-2 Assay | Two regions in orf1ab | Hologic |
| BD SARS-CoV-2 Reagents for the BD Max System | N1 and N2 regions | Becton Dickinson |
| BioFire Respiratory Panel 2.1 | S and M genes | bioMérieux |
| E gene laboratory-developed test (LDT) | E gene | 1 |
| SARS-CoV-2/FluAB LDT | E gene | 2 |

**Table S2. Oligonucleotides used in the Omicron assays.**

| **Oligonucleotide** | **Sequence** | **Concentration** | **Reference and notes** |
| --- | --- | --- | --- |
| E gene  COVID19_E_For_V2  COVID19_E_Rev_V2  COVID19_ E_MGB_NED | 5’- GAG ACA GGT ACG TTA ATA GTT AAT AGC G -3’  5’- CAA TAT TGC AGC AGT ACG CAC AC -3’  5’- CTA GCC ATC CTT ACT GCG -3’ (NED/MGB) | 0.8 μM  0.8 μM  0.2 μM | 1  PCR efficiency: 99.4% |
| MS2  MS2-TM2-For  MS2-TM2-Rev  MS2-TM2_ATTO647 | 5’- TGC TCG CGG ATA CCC G -3’  5’- AAC TTG CGT TCT CGA GCG AT -3’  5’- ACC TCG GGT TAO TTC CGT CTT GCT CGT -3’ (ATTO647/Iowa Black) | 0.72 μM  0.72 μM  0.1 μM | 3  PCR efficiency: Not determined |
| S:N501Y  Covid_SpkN501Y_For2  Covid_SpkN501Y_Rev  Covid_Spk_N501Ymutant_PHOLv2_FAM_BHQ1 | 5’- ACA CCT TGT AAT GGT GTT GMA GG-3’  5’- AGT TGC TGG TGC ATG TAG AAG TTC -3’  5’- CCR ACC CAC TTA TGG TGT TG-3’ (FAM/BHQ) | 0.8 μM  0.8 μM  0.07 μM | 4, PHOL*  PCR efficiency: 102.8% |
| S:ins214EPE  Covid_Spk214EPE_For  Covid_Spk214EPE_Rev  Covid_Spk214EPE_HEX | 5’ – AAA TAT ATT CTA AGC ACA CGC CT – 3’  5’ – GGC AAA TCT ACC AAT GGT TCT AAA G – 3’  5’ – GCG TGA GCC AGA AGA TCT CC – 3’ (HEX/BHQ-1) | 0.8 μM  0.8 μM  0.2 μM | This study  PCR efficiency: 102.1% |
| S:H69/V70  Covid_Spk69/70_For  Covid_Spk69/70_Rev  Covid_Spk69/70_WT_Fam_MGB | 5’- AGT TTT ACA TTC AAC TCA GGA CTT GTT C -3’  5’- GAC AGG GTT ATC AAA CCT CTT AGT ACC -3’  5’- CAT GCT ATA CAT GTC TCT GG -3’ (FAM/NFQ-MGB) | 0.8 μM  0.8 μM  0.2 μM | 5  PCR efficiency:  99.1% |
| orf7b:L11F  Covid_Orf7b_L11F_For  Covid_Orf7b_L11F-Rev  Covid_Orf7b_L11F_Probe-VIC  Covid_Orf7b_L11F_Probe-HEX | 5’ – TTA TAA CAC TTT GCT TCA CAC TC – 3’  5’ – GAT AAT AAG CAT AAT TAA AAC AAG G– 3  5’ – GAC TTC TAT TTT TGC TTT TTA GCC– 3’ (VIC/BHQ-1)  5’ – GAC TTC TAT TTT TGC TTT TTA GCC– 3’ (HEX/BHQ-1) | 0.8 μM  0.8 μM  0.2 μM  0.2 μM | This study  HEX probe used Jul 19-Nov 22, 2022, VIC used Nov 23-Dec 31, 2022  PCR efficiency: 102.5% |
| M:D3N  Covid_M_D3N_For  Covid_M_D3N_Rev  Covid_M_D3N_Probe-NED-MG | 5’ – TGA TCT TCT GGT CTA AAC GAA C – 3’  5’ – CTT TTT AAG CTC TTC AAC GG – 3’  5’ – CAT GGC AAA TTC CAA CGG – 3’ (NED/MGB-NFQ) | 0.5 or 0.8 μM  0.5 or 0.8 μM  0.13 or 0.2 μM | This study  The lower concentrations used Jul 19-Nov 22, 2022, the higher concentrations used Nov 23-Dec 31, 2022  PCR efficiency:  93.9% |

*PHOL: Public Health Ontario Laboratory

**References**

1. Pabbaraju, K. *et al*. Development and validation of RT-PCR assays for testing for SARS-CoV-2. *J. Assoc. Med. Microbiol. Infect. Dis. Can.* **6(1)**, 16-22 (2021).

2. Pabbaraju, K., Wong, A. A., Ma, R., Zelyas, N. & Tipples, G.A. Development and validation of a multiplex reverse transcriptase-PCR assay for simultaneous testing of influenza A, influenza B and SARS-CoV-2. *J. Virol. Methods.* **293**, 114151 (2021).

3. Dreier, J., Störmer, M. & Kleesiek, K. Use of bacteriophage MS2 as an internal control in viral reverse transcription-PCR assays. *J. Clin. Microbiol.* **43(9)**, 4551-7 (2005).

4. Pabbaraju, K. *et al*. Evolving strategy for an evolving virus: Development of real-time PCR assays for detecting all SARS-CoV-2 variants of concern. *J. Virol. Methods*. **307**, 114553 (2022).

5. Zelyas, N. *et al*. Precision Response to the Rise of the SARS-CoV-2 B.1.1.7 Variant of Concern by Combining Novel PCR Assays and Genome Sequencing for Rapid Variant Detection and Surveillance. Microbiol. Spectr. **9(1)**, e0031521 (2021).
